# Impact of molecular structures of lauroyl glycine derivatives on foaming properties

**DOI:** 10.3389/fchem.2025.1563560

**Published:** 2025-06-24

**Authors:** Rourou Li, Guanjun Zhang, Shuyan Yu, Ruyv Deng, Jiechun Yin, Taijun Zhang, Qiuxing He

**Affiliations:** ^1^ School of Chemical Engineering, Guangdong Pharmaceutical University, Guangzhou, China; ^2^ Technical Support Department, Emperor’s Wisdom Skin and Biotechnology Institute, Guangzhou, China; ^3^ School of Materials and Chemical Engineering, Zhengzhou University of Light Industry, Zhengzhou, China

**Keywords:** foam, surfactant, cleaning, amino - acid, sebum

## Abstract

**Introduction:**

This investigation systematically elucidates the foam dynamics and consumer perception correlations within amino-acid-derived surfactant-mixedcomponent systems.

**Methods:**

pH-gradient experiments (5.5–10) combined with dynamic foam analysis were employed to quantify foam nucleation kinetics. Molecular dynamics simulations analyzed intermolecular interactions, while lipid resistance evaluations measured cleaning efficiency.

**Results:**

SLG-CAB blends accelerated foam nucleation from 35 s to 20 s/100 mL (pH=8.5), outperforming commercial benchmarks (<5 s initial formation) with statistical significance. Robust hydrogen bonds between CAB’s ammonium protons and SLG’s carboxamide oxygen (bond length: 1.901 Å) achieved thermodynamic stabilization (ΔE = –53.04 kcal/mol), enhancing film stability (Tfls 50% > 5 min). SLG-CAB generated monodisperse bubbles (diameter ≈95 μm), imparting “velvety” sensory properties, with 74.74% lipid cleaning efficiency at pH 7–8 (synergistic coefficient βs = –2.822).

**Discussion:**

The SLG-CAB system demonstrates synergistic foam enhancement and lipid resistance, enabling “prolonged creaminess” in cleansing applications. Bridging cosmetic applications (facial cleansers, body washes) with surfactant engineering principles, this work establishes phase behavior-guided formulation strategies for personal care products.

## 1 Introduction

Foam serves as a critical sensory indicator for consumers to evaluate cleansing efficacy in personal care products. Market analyses reveal that 67% of users associate “rich lather” with product effectiveness, while “slow foaming” negatively impacts satisfaction even if cleaning capacity is comparable (Zhang Y. et al., 2023). Despite this psychological linkage, the scientific basis connecting molecular structure, foam dynamics (bubble size, formation kinetics), and sebum removal remains unclear, particularly for amino acid-based surfactants.

Foam’s metastable gas-liquid structure enables dual functions in cleansing: (i) the high surface area facilitates sebum emulsification, and (ii) shear-thinning rheology allows physical removal of debris from skin folds (Yang et al., 2023). However, achieving optimal foam properties requires precise control over bubble size (<95 μm for “silky” texture) and drainage stability (>5 min T_fls_ 50% for sustained application) (Sheng et al., 2023). Traditional surfactants like Lauric acid (Soap-based surfactants) generate abundant foams but often compromise epidermal integrity due to high alkalinity (pH 9‒10) and lipid bilayer disruption (Cohen-Addad et al., 2013) (Boos et al., 2013). In contrast, amino acid surfactants (pH 5.5‒6.5) mimic the skin’s acidic mantle, reducing irritation risks. Yet, their foaming capacity and grease resistance lag behind harsh detergents, limiting commercial adoption.

Sebum—a lipid mixture of triglycerides, squalene, and cholesterol—forms both a protective barrier and a breeding ground for pathogens when overaccumulated (Rosik et al., 2024). While surfactants lower sebum-water interfacial tension to enable removal, excessive defatting disrupts the stratum corneum, triggering dryness and inflammation (Landemaine et al., 2023). Lauric acid (LA)-derived surfactants exemplify this trade-off: their strong alkalinity (pH 9.3 ± 0.2) and small headgroups confer high grease solvency but damage keratinocyte (Grassia, 2021). Amino acid surfactants,such as sodium lauroyl glycinate (SLG) and sodium lauroyl sarcosinate (SLS), consist of amino acid-derived hydrophilic head groups and hydrophobic chains, with the head group structure playing a critical role in their interactions with other substances through hydrogen bonding and electrostatic interactions (Salomon and Giordano-Labadie, 2022). However, the impact of these structural nuances (e.g., amide orientation, methyl branching) on foam-sebum interactions remains undefined.

Current strategies to optimize amino acid surfactants focus on monomeric properties like critical micelle concentration (CMC) or skin tolerance thresholds (Tackie-Otoo et al., 2022). The admixture with secondary surfactants is an established industrial practice aimed at enhancing foam performance through synergistic effects, yet the delipidation mechanisms remain less explored. For instance, while amphoteric Cocamide propyl betaine (CAB) may stabilize amino-acid-based surfactants via charge neutralization (Bae et al., 2021), its regulatory effects on foam-sebum interfacial mechanics have been largely overlooked in prior investigations (Preisig et al., 2019; Li et al., 2023). This study systematically evaluates the foam dynamics and lipid resistance of betaine/sulfonate/sulfate-headed surfactants formulated with amino-acid-based counterparts (Wu et al., 2024; Shahmarvand et al., 2023).

### 1.1 Innovation and objectives

We establish quantitative correlations between molecular features (amide groups, chain branching), objectively measured foam properties (bubble size, rise velocity), and consumer sensory descriptors (e.g., “density,” “speed”). Through dynamic simulations, we reveal how amide groups in SLG/SLS form ordered hydrogen-bond networks at foam lamellae, resisting sebum penetration without sacrificing foamability. By combining amino acid surfactants with CAB/AES/SMCT, we demonstrate charge-complementary and hydrogen-bond-mediated stabilization mechanisms inaccessible to single-component systems. Contrasting with LA benchmarks (pH 9.5), our SLG-CAB blends achieve pH 7-8 while maintaining >70% sebum removal efficiency. Such pH-adjusted systems promise to redefine “gentleness” in cleansers by preserving both skin barrier function and user-perceived efficacy.

### 1.2 Experimental design

**FIGURE 1:**
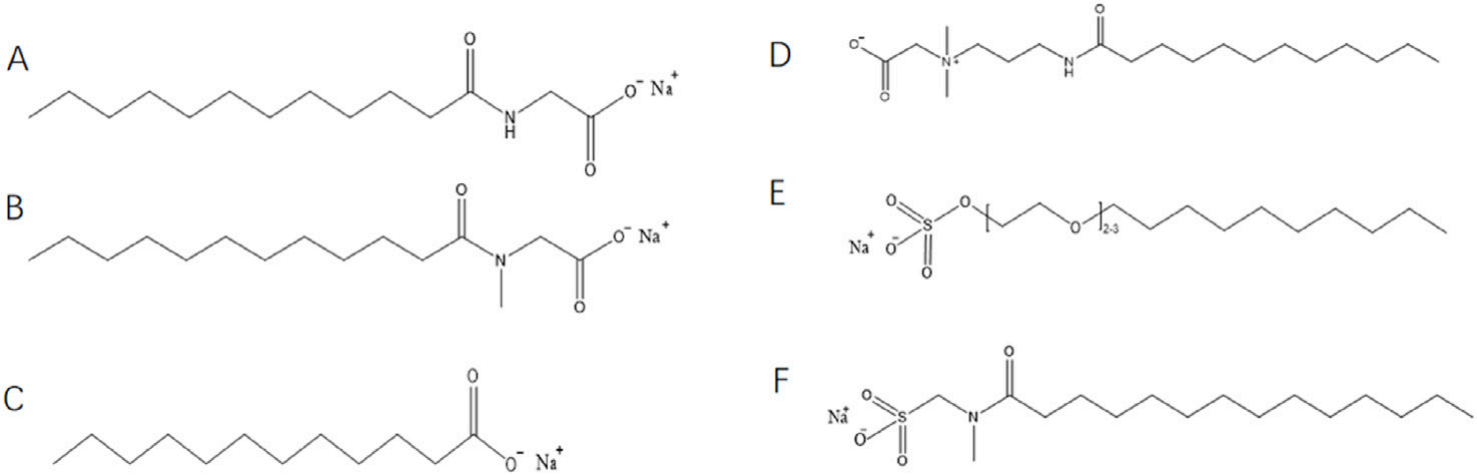
Chemical structural formula for surfactant molecules: **(A)** Sodium lauroyl glycinate, **(B)** Sodium lauroyl sarcosinate, **(C)** Lauric acid, **(D)** Cocamide propyl betaine, **(E)** Sodium lauroyl ether sulfate **(F)** Sodium methyl cocoyl taurate.

We selected SLG (amide), SLS (branched amide), and LA (amide-free) to isolate the role of amide functionality. The chemical structure is shown in Figure 1. These were blended with CAB, AES, or SMCT at fixed ratios (2:1 wt%). Foam dynamics were quantified using a Krüss DFA100 analyzer, with lipid resistance tested via artificial sebum simulating human sebaceous composition. Molecular dynamics (MD) simulations deciphered hydrogen-bonding patterns and interfacial self-assembly.

## 2 Material and methods

### 2.1 Materials and reagents

The following surfactants were used in this study: Sodium lauroyl sarcosinate (≥90%), Sodium lauroyl glycinate (≥90%), Lauric acid (≥90%), and Sodium methyl cocoyl taurate (≥90%), all of which were procured from Nanjing Huashi New Material Co., Ltd. Cocamide propyl betaine (≥90%) was purchased from Guangzhou Flower’s Song Co., Ltd., and Sodium lauroyl ether sulfate (≥90%) was obtained from Hunan Resun Co., Ltd.

### 2.2 Mixed system preparation

Preparing the mixed systems, Sodium lauroyl sarcosinate (SLS), Sodium lauroyl glycinate (SLG), and Lauric acid (LA) were combined with Sodium methyl cocoyl taurate (SMCT), Cocamide propyl betaine (CAB), and Sodium lauroyl ether sulfate (AES) in various formulations. The total surfactant concentration in all prepared sample solutions was maintained at 0.5 wt%. The primary surfactant to co-surfactant formulation ratio was maintained at 2:1.

### 2.3 Surface tension

Surface tension measurements were performed using the Du Noüy ring method on a K12 Tensiometer (Krüss, Germany) at 25°C and atmospheric pressure (101.325 kPa). Prior to each measurement session, the platinum-iridium ring (circumference 6 cm) was calibrated with a 500 mg standard weight following ISO 304:2021 guidelines (Emami et al., 2022). This calibration ensured force measurement accuracy of ±0.01 mN/m. Before sample testing, the ring was sequentially cleaned by:

a) Flame sterilization at 300°C for 10 s
b) Solvent rinse (ethanol:water = 7:3 v/v)
c) Ultrapure water (18.2 MΩ cm) immersion
d) Repetitive blank tests until baseline consistency (±0.2 mN/m)

Each sample solution (n ≥ 3 replicates) was measured with fresh aliquot,the standard deviation across replicates was maintained at ≤0.1 mN/m (Wang et al., 2022).

### 2.4 Foam production and characterization

The foam properties were analyzed using a dynamic foam analyzer (DFA100, Kruss GmbH). The analyzer consists of a foam column, foam base column, luminizer, and light detector. The gas phase above the foam and liquid is transparent, while the foam column absorbs part of the emitted light. The system detects two-phase boundaries—liquid/foam and foam/air—by measuring differences in transmittance. The optical detection recorded the following parameters:

(1) Foam bubble size and its statistical distribution,
(2) Liquid volume within the foam, and
(3) Maximum foam volume.

All measurements were conducted at a temperature of 25°C and atmospheric pressure. For each test, 40 mL of the sample solution was used. The rotation speed was set to 4000 RPM, with a rotation time of 48 s, and foam stability was observed over a 12-min duration (Xue et al., 2018).

### 2.5 Rheological properties test

The rheological properties of foam, including elasticity and viscosity, play a crucial role in determining its performance in various applications. In this study, the rheological behavior of foam stabilized with a mixed amino surfactant system was investigated. Rheological measurements were performed using an Anton Paar MCR102 rheometer. The tests were conducted using a plate geometry and a PP50 rotor. The temperature was maintained at 25°C throughout the measurements, and the test gap was set to 1 mm. The viscoelastic modulus of the foam in the mixed amino surfactant system was assessed at a fixed frequency of 10 rad/s, with a strain range spanning from 0.01% to 100%.

### 2.6 Foam oil resistance test

The sebum sample was prepared in the laboratory, and the configuration method was referred to (Mijaljica et al., 2024). Two ways to measure foam cleaning capacity:

#### 2.6.1 Degreasing capacity of unformed foam

To evaluate the degreasing capacity of unformed foam, 40 mL of the solution sample and 0.5 mL of sebum sample were added into the DFA100 glass column. The rotation speed was set to 4000 RPM, and the rotation time was maintained at 48 s. After foaming, the foam height was observed immediately. This method assesses foaming by emulsifying a small amount of oil, which is more representative of the conditions encountered during typical body wash usage.

#### 2.6.2 Degreasing ability of formed foam

To measure the degreasing ability of formed foam, a red oil-soluble dye was used to stain the oil for easy visualization. A specific amount of oil was applied to a glass plate, and foam was sprayed onto the plate. The change in the oil area was recorded using a camera. After the cleaning process, the foam was removed, and the glass plate was dried and weighed. The cleaning efficiency was calculated using the following formula. This setup simulates the process of cleaning with formed foam, akin to typical cleaning scenarios.

$$Cleaning\;efficiency\;\%=\left(m_{1}-m_{2}\right)/\left(m_{1}-m_{0}\right)*100\%$$

where m_0_ is the initial weight of the soiled glass plate; m_1_and m_2_ are the weights of the contaminated glass plate before and after cleaning, respectively (Li et al., 2024).

### 2.7 Molecular dynamic simulation

The classic sandwich model (Du et al., 2025) was established using the packmol (Martínez et al., 2009) software. The key to the model is the construction of the contact surface between the layers and the surfactant layer and the water molecule layer. The polar heads of the surfactant molecules must be closely packed to prevent water molecules from overflowing or even the foam from breaking. The Amber99 molecular force field was adopted for the system, and the simulation temperature was set at 298K. Firstly, the system energy minimization (2 ns) was carried out to eliminate the unreasonable short distances between atoms in the system. Then, the NPT (2 ns) restricted simulation was conducted, followed by a 10 ns unrestricted dynamic simulation. The simulation process was completed by the Yasara (Land and Humble, 2018) software, and the final analysis and plotting were performed using the VMD 1.9.3 software (Humphrey et al., 1996) and PyMol (The PyMOL Molecular Graphics System, Version 1.7, Schrodinger, LLC.) software.

## 3 Results and discussion

### 3.1 Foam dynamic behavior

#### 3.1.1 Foamability: foaming speed and foam height

During consumer product usage, both foaming speed and foam volume significantly impact user experience. Slow foaming speed and insufficient foaming quantity are perceived as indicators of inferior product quality. Therefore, sufficient and abundant foam must be generated within an extremely short timeframe (5 s, given typical handwashing duration of 10–20 s). Both parameters require systematic evaluation. pH range selection: Experimental observations revealed that SLS precipitates at pH 5.5 due to carboxyl group protonation, while SLG precipitates at pH 7 and LA at pH 9 (Towesend et al., 2022). Notably, SLS exhibited markedly reduced foaming performance at pH 8. The operational pH window was therefore established as 5.5–8. Although cosmetic formulations (e.g., foam depilatories) may extend to pH 12, the upper limit was cautiously set at pH 10 to minimize dermal irritation (Mu et al., 2023).

Through solubilization enhancement by auxiliary surfactants, all three primary surfactants maintained stability without precipitation under reduced pH gradients (ΔpH = 1–0.5). Subsequent pH adjustments at 0.5 intervals enabled comparative analysis of foam formation characteristics across mixed systems.

**FIGURE 2:**
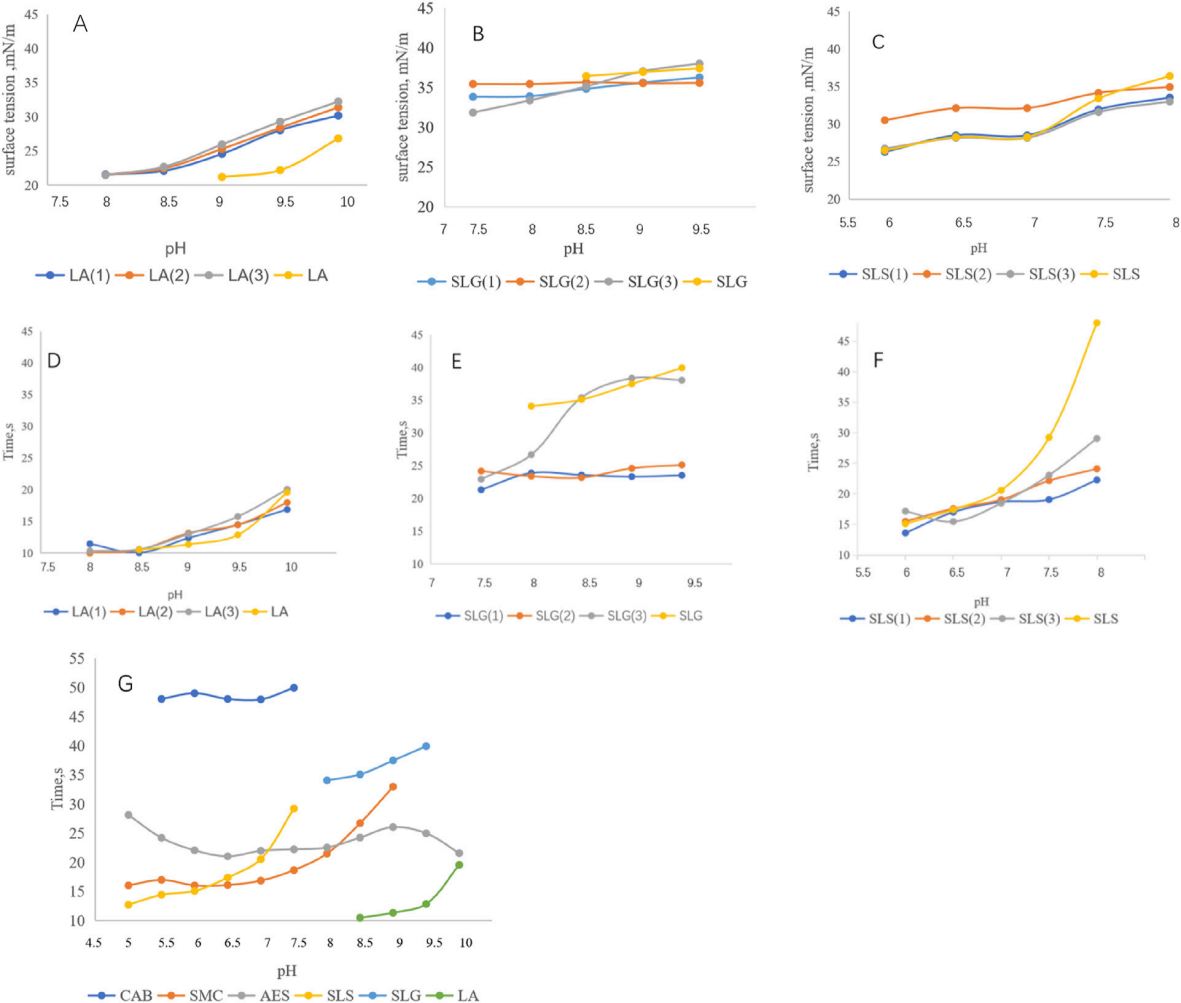
**(A–C)** Surface tension of each series at different pH: **(A)** LA (1): LA-CAB system、LA (2): LA-AES system, LA (3): LA-SMCT system. **(B)** SLG (1): SLG-CAB system, SLG (2) SLG-AES system, SLG (3): SLG-SMCT system. **(C)** SLS (1): SLS-CAB system, SLS (2): SLS-AES system, SLS (3): SLS-SMCT system. LA series demonstrate surface tension ranges of 21–32 mN/m; SLG series exhibit a narrower variation range of 32–36 mN/m; SLS series show wider fluctuations spanning 25–36 mN/m Experimental conditions: 25°C; measurements were performed in triplicate using a surface tension meter. The obtained values followed a normal distribution, and the mean value was adopted. **(D–F)** Different series and Pure products of foaming speed at different pH,Time/100 mL: Experimental conditions: 25°C; the foaming solution volume was 40 mL, and the time required for the foam volume of each solution to reach 100 mL was recorded. The LA series achieves 100 mL foam volume within 20 s, while the SLG series requires 20–30 s and the SLS series within 15–25 s in foaming speed quantification. **(G)** Pure products of foaming speed at different pH.

Figure 2 demonstrates the correlation between surface tension and foaming kinetics (time to reach 100 mL foam volume). The LA series exhibited surface tension values of 20–30 mN/m with remarkably rapid foaming (10–20 s to 100 mL) (Figures 2A,D). For SLG systems, auxiliary surfactants showed limited capacity in surface tension reduction (-2 mN/m) Figures 2B,C, yet significantly enhanced foaming speed from 35s/100 mL to 20 s/100 mL at pH 8.5 (Varade and Ghosh, 2020; Vu et al., 2020). These suggest their role in mitigating headgroup electrostatic repulsion and/or improving molecular mobility. Conversely, SLS systems showed no surface tension reduction with auxiliary surfactants-the sulfonic acid groups even increased surface tension. However, under neutral conditions (pH 7–8), accelerated foaming speed was observed. These findings demonstrate that while the synergetic interaction exerted no measurable effects on surface tension modulation, it effectively facilitated enhancement of foaming speed. Figures 2E,F highlight CAB as the most effective auxiliary surfactant for enhancing foaming speed in both SLS and SLG systems.

**FIGURE 3:**
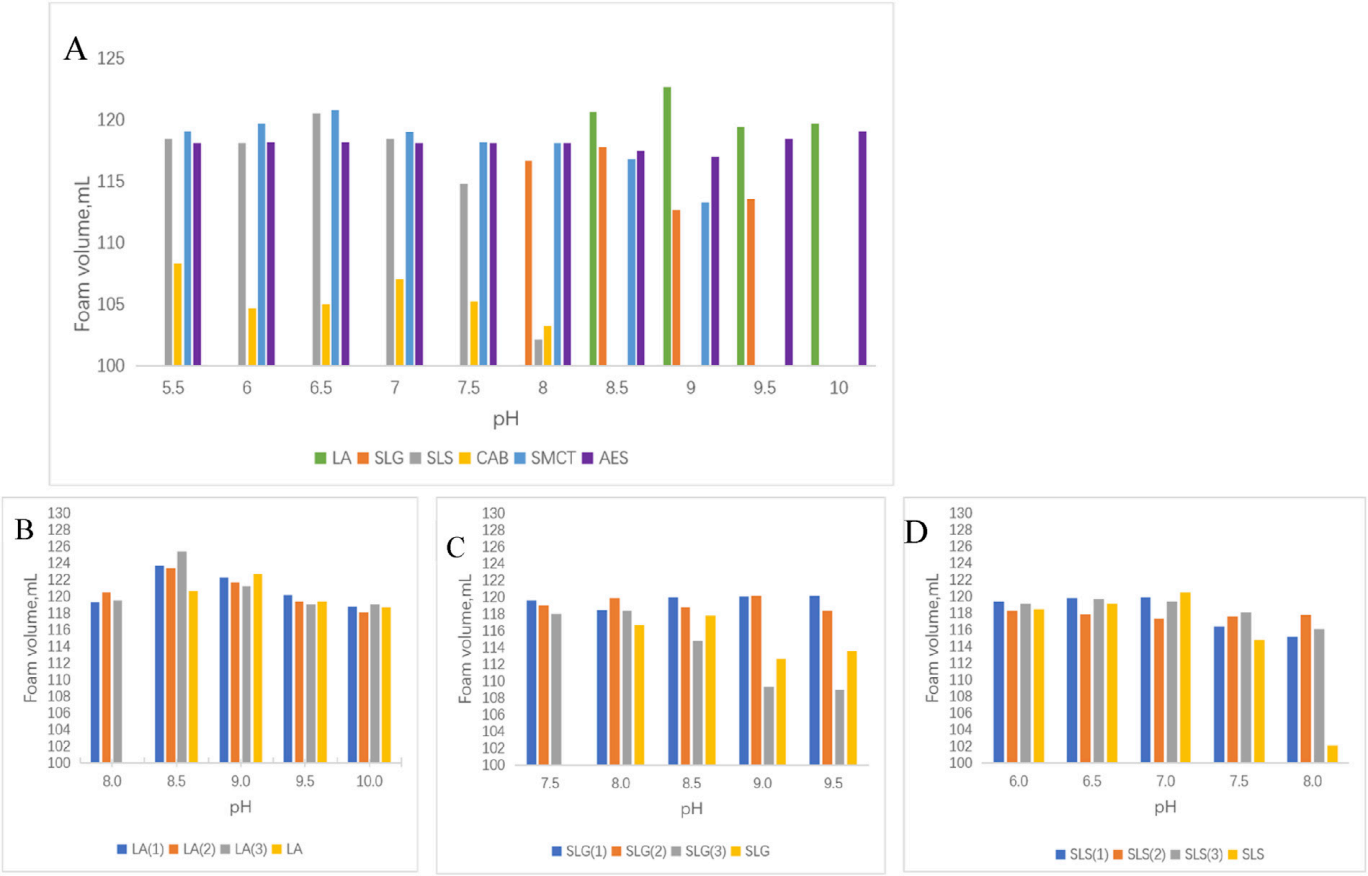
**(A)**: Pure products of Foaming volume at different pH. **(B–D)** Foaming volume of each series: **(B)** LA (1): LA-CAB system, LA (2): LA-AES system, LA (3): LA-SMCT system. **(C)** SLG (1): SLG-CAB system, SLG (2): SLG-AES system, SLG (3):SLG-SMCT system. **(D)** SLS (1): SLS-CAB system, SLS (2): SLS-AES system, SLS (3): SLS-SMCT system. In pH-dependent evaluations, the CAB surfactant exhibits foam volume below 108 mL, while other surfactants demonstrate values exceeding 112 mL. At pH 8.5, LA-SMCT achieves 124 mL foam height. SLG-CAB/SLG-AES systems maintain pH-stable foaming capacity at 119 ± 1 mL. Under pH 7 conditions, SLS series surfactants deliver optimal performance with foam volumes consistently above 118 mL. Experimental conditions: 25°C; the foaming solution volume was 40 mL with a foaming duration of 48 s. Recording the total foam volume after completing the foaming process.

Foam volume analysis (Figure 3) revealed strong pH dependence for amino acid-based and zwitterionic surfactants, contrasting with AES and LA systems. This phenomenon may arise from carboxyl group protonation/deprotonation processes near the pKa of amino acid surfactants (Zhang et al., 2016). Notably, individual CAB and SLS solutions at pH 8 produced ≤105 mL foam, yet their combination achieved 116 mL. Pure CAB formulations averaged 105 mL foam volume, but synergistic enhancement occurred when combined with primary surfactants. We hypothesize that betaine-amide group interactions might facilitate hydrogen bond formation, thereby improving foam stability and gradually increasing foam volume during expansion. Sulfonic acid groups displayed divergent effects: suppressive for SLG yet cooperative for SLS. Potential mechanisms include disruption of SLG-water hydrogen bonds by sulfonic acid groups or alkaline-induced destabilization of sulfonic acid-based foam architectures.

Intriguingly, while amide group incorporation improved cutaneous mildness, it correspondingly reduced maximum foam volume to 116 mL (pH 8.5). The introduction of branched-chain moieties coincided with an optimal pH shift to 6.5, achieving a foam volume of 120 mL—demonstrating striking effectiveness under the specified conditions.

#### 3.1.2 Bubble size

Foam bubble size significantly influences consumer perception of foam density and loftiness. Compact foams demonstrate enveloping characteristics, providing cloud-like textural density in facial mousse applications, while fluffy foams convey sensory comfort for relaxation in bubble bath products. The evaluation of bubble size merits context-specific analysis rather than absolute characterization. For facial cleansing formulations (typically applied ≤3 min), maintenance of foam volume and bubble diameter stability becomes particularly critical for body mousse or depilatory mousse applications requiring 10–12 min contact duration, necessitating systematic investigation of foam stability.

**FIGURE 4:**
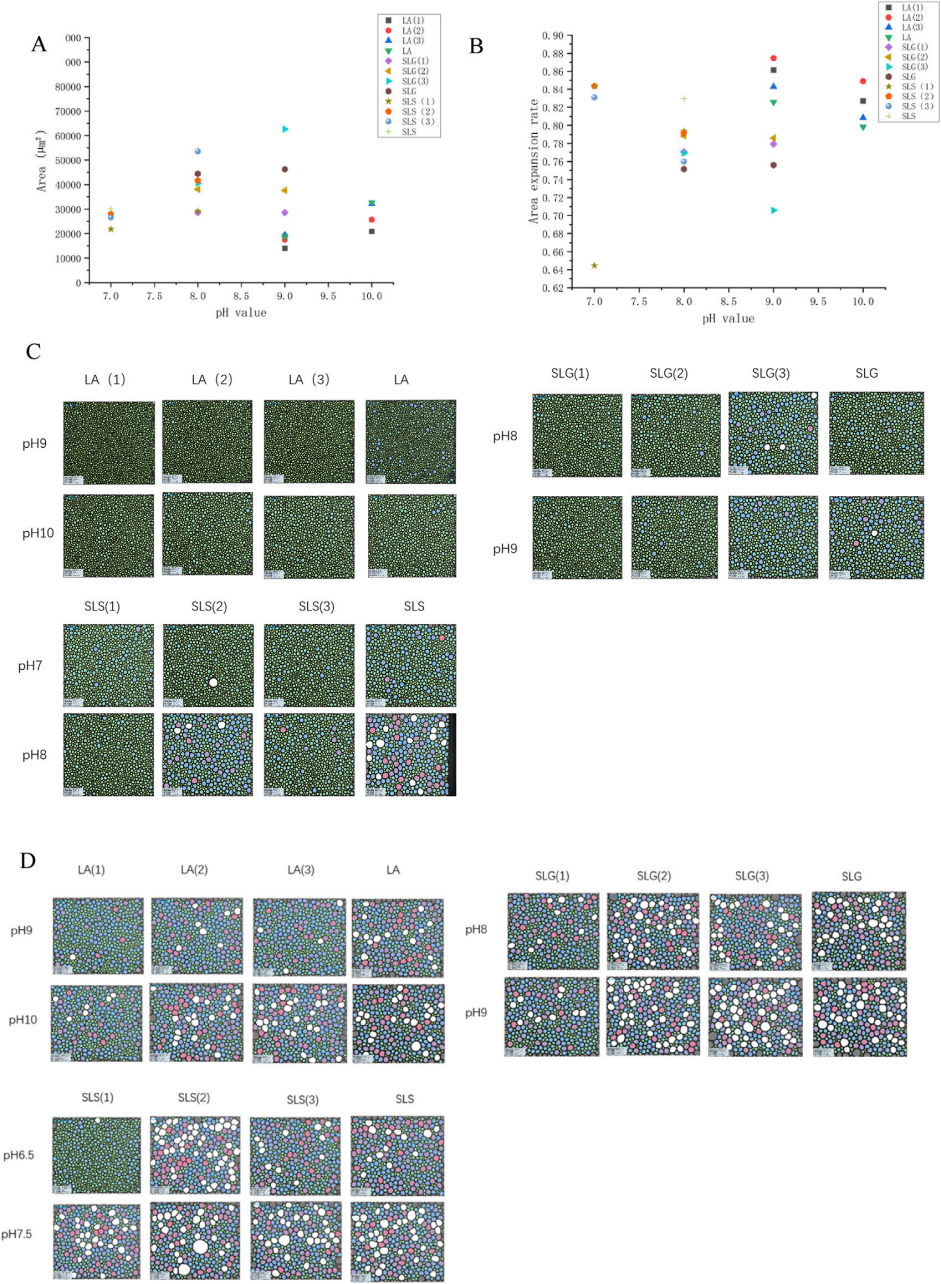
The bubble dimensions was monitored at three time intervals: 0 s (immediately post-foaming), 6 min, and 12 min after the foaming process. **(A)** Average area of initial bubble: LA (1): LA-CAB system, LA (2): LA-AES system, LA (3): LA-SMCT system.; SLG (1): SLG-CAB system, SLG (2): SLG-AES system, SLG (3): SLG-SMCT system; SLS (1): SLS-CAB system, SLS (2): SLS-AES system, SLS (3): SLS-SMCT system; The LA-CAB system displays minimum initial foam dimensions of 13,940 μm^2^ at pH 9.0 across all tested series. Comparative analysis reveals SLG-CAB achieves 28,642 μm^2^ at pH 8.0, while SLS-CAB exhibits its minimal foam volume of 21,799 μm^2^ under pH 7.0 conditions. [B]Bubble area expansion ratio ((average bubble area at 12 min - average bubble area at 6 min)/average bubble area at 12 min); **(C)** The bubble area at 6 min **(D)** The bubble area of the bubble at 12 min.

Figure 4A demonstrates the LA series exhibited microbubble radii 90 <μm, contrasting with other systems showing initial bubble radii of 95–130 μm. This size discrepancy may correlate with molecular dimensions and headgroup electrostatic repulsion. Geometric mean radius calculations yielded 3.649 Å for LA, 4.042 Å for SLG, and 4.119 Å for SLS. Assuming constant repulsive forces, smaller molecular dimensions facilitate tighter packing alignments, consequently favoring the formation of bubbles with reduced radii. Auxiliary surfactants manifested differential effects: CAB and AES universally reduced bubble size, while SMCT decreased bubble radius in SLS systems but increased it for SLG and LA. Notably, SMCT and CAB possess matching geometric radii (∼3.480 Å), suggesting that headgroup charge characteristics dominate bubble size modulation, as evidenced by comparative analysis in Figures 4A,B. Comparative analysis of Figures 4C,D reveals progressive foam expansion through concurrent Ostwald ripening (bubble coalescence) and foam collapse during the 6–12 min timeframe. Bubble expansion rates were quantitatively compared in Figure 4B: SLS(1) maintained visibly smaller bubble radii at 12 min with only 64% expansion, other systems exhibited 70%–90% expansion. However, auxiliary surfactants failed to suppress bubble expansion (coalescence) in LA systems. Intriguingly, SLG(3) under alkaline conditions demonstrated effective expansion inhibition. This unexpected behavior suggests that sulfonic acid groups, while ineffective in foam generation, may contribute to foam stability maintenance.

#### 3.1.3 Foam stability

**FIGURE 5:**
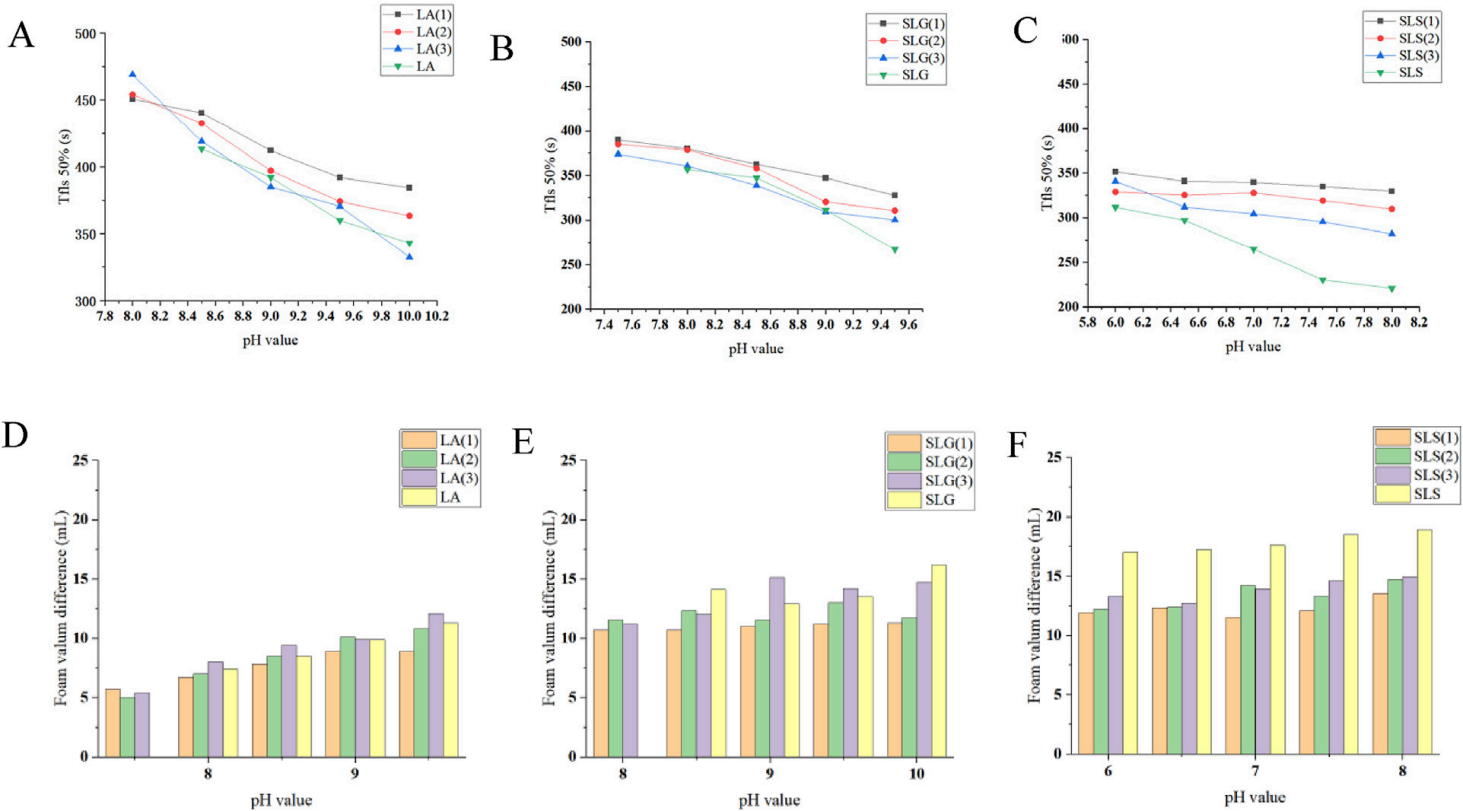
**(A–C)** Pure products and different series of T_fls_ 50% (The time required for the solution volume to return to 50% of its original value after foam formation.): **(A)** LA (1): LA-CAB system, LA (2): LA-AES system, LA (3): LA-SMCT system. **(B)** SLG (1): SLG-CAB system, SLG (2): SLG-AES system, SLG (3): SLG-SMCT system. **(C)** SLS (1): SLS-CAB system, SLS (2): SLS-AES system, SLS (3): SLS-SMCT system; Maximum and minimum values for each series: LA-SMCT: 468.9 s (pH 8.0) at T_fls_ 50% (peak) → 332.4 s (pH 10.0) at T_fls_ 50% (minimum); SLG-CAB/SLG: 390.0s (pH 7.5) at T_fls_ 50% (peak) → 267.1 s (pH 9.5) at T_fls_ 50% (minimum); SLS-CAB/SLS: 351.4 s (pH 6.0) at T_fls_ 50% (peak) → 220.9 s (pH 8.0) at T_fls_ 50% (minimum). **(D–F)** Pure products and different series of Foam volume difference (△V=V _Initial_ -V_at 12 min_) at different pH: The LA series demonstrates the smallest foam volume variation among the three tested systems, with differences consistently below 12 mL. SLG-CAB maintains stable performance with minimal volume fluctuations (∼11 mL). Notably, SLG-SMCT exhibits progressive variance expansion (>pH 9.0 conditions). Singular SLS solutions show the most pronounced volumetric instability, exceeding 16 mL across all test pH.

Foam stability was also evaluated by determining the T_fls_50% (time required for 50% foam volume reduction) and foam height differences, providing further insight into the foaming behavior (Figure 5).

Foam stability is primarily determined by the thickness of the liquid film and the coalescence of foam (Kang et al., 2023). In this experiment, the maximum detection time was set at 12 min foam stability was quantitively assessed through conjoint analysis of T_fls_ 50% and volumetric differentials between metastable and equilibrated foam columns. Analytical data revealed statistically superior stability in LA-stabilized systems compared to SLS/SLG system (p < 0.01). This phenomenon is mechanistically attributed to electrosteric stabilization effects induced by carboxamide-functional groups, as evidenced by colloidal potential characteristics: SLG (−85.96 mV), SLS (−67.52 mV) versus LA (−32.34 mV) (Mijaljica et al., 2024). The higher repulsion in SLG foam reduces the membrane stability compared to LA. SLG can form hydrogen bonds, leading to a stronger film compared to SLS (Li et al., 2024).

CAB demonstrates superior foam stability, which may be due to its amphoteric nature. In non-ideal conditions, CAB molecules in the foam film can arrange with their cationic and anionic heads close to one another, creating adsorbing forces on the foam surface. These forces weaken the electrostatic repulsion of the head groups, increase film strength, and delay foam rupture. Additionally, CAB exhibits a stronger synergistic effect with other surfactants (as discussed in Sections 3.1.2–3.1.3), which enhances water retention and slows foam drainage (Zhu and Zheng, 2023).

### 3.2 Foam mechanism analysis

#### 3.2.1 Rheological properties

Foam viscosity and elasticity critically determine tactile perception characteristics (Zhu and Zheng, 2023). Elevated viscosity correlates with cohesive matrix retention, markedly reducing collapse propensity, whereas enhanced elasticity enables three-dimensional structural integrity and shape adaptability. Empirical evidence demonstrates that rigid, densely packed foams with well-defined lthree-dimensional architecture exhibit statistically significant preference increments in consumer acceptability assessments.

The rheological properties of foam are critical as they directly influence foam stability, which is closely related to the Ostwald maturation and drainage processes. The fluidity of the liquid surrounding the foam bubbles is primarily determined by viscosity. In the loss modulus curve of all foams, a gradual decrease is observed initially, followed by a rapid decline after reaching a certain shear strain. This point represents the yield point of the foam (Sheng et al., 2023). The foam yield point indicates the shear strain required to overcome the structural integrity of the foam, causing adjacent bubbles to separate and slide past each other. When comparing the pure surfactant systems with the mixed systems, it is evident that the yield point in the mixed system shifts to a higher strain, and the viscosity increases. This change effectively reduces drainage and gas diffusion, as the more viscous foam exhibits greater resistance to these processes (Yang and Pal, 2020).

**FIGURE 6:**
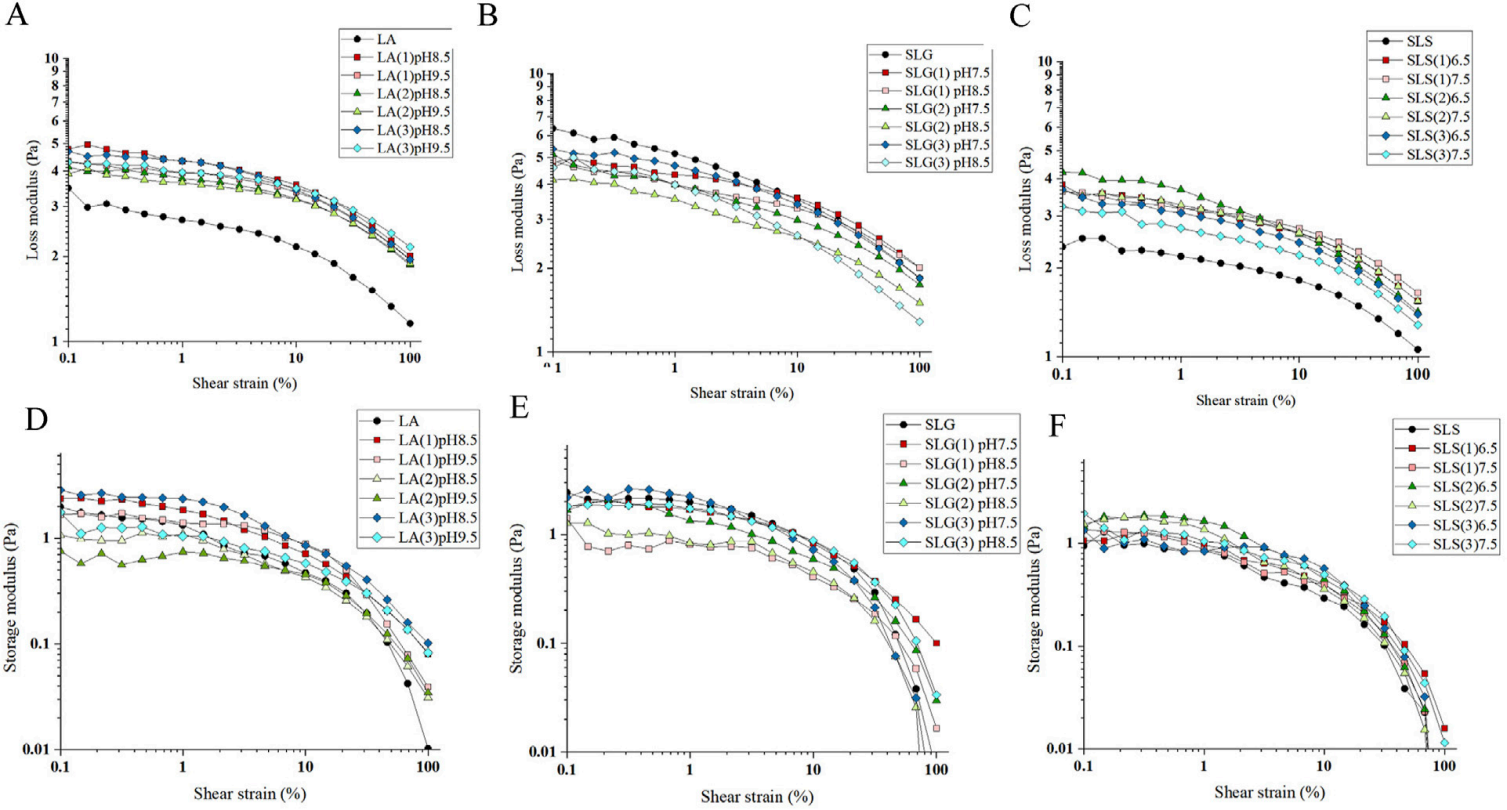
Experimental conditions: Anton Paar MCR102 rheometer; 25°C; The tests were conducted using a plate geometry and a PP50 rotor.; Test gap was set to 1 mm. The viscoelastic modulus of the foam in the mixed amino surfactant system was assessed at a fixed frequency of 10 rad/s, with a strain range spanning from 0.01% to 100%. **(A-C)** Pure products and different series of Loss modulus at different pH: **(A)** LA (1): LA-CAB system, LA (2): LA-AES system, LA (3): LA-SMCT system. **(B)** SLG (1): SLG-CAB system, SLG (2): SLG-AES system, SLG (3): SLG-SMCT system. **(C)** SLS (1): SLS-CAB system, SLS (2): SLS-AES system, SLS (3): SLS-SMCT system. **(D–F)** Pure products and different series of Storage modulus at different pH.

The foam rheological properties were further explored through oscillatory experiments. The primary factors affecting the elasticity of foam are gas content and bubble size. A denser foam with smaller bubble sizes typically exhibits higher elasticity and greater yield stress. As shown in Figures 4, 6, the LA hybrid system, which has the smallest foam size, demonstrates superior initial deformation resistance compared to the other systems. Interestingly, SMCT also performs well in terms of deformation resistance, whereas CAB shows no significant improvement in this aspect.

#### 3.2.2 Interaction parameters

According to the mixed adsorption theory proposed by Rosen et al. (Liu et al., 2021), binary mixtures of surfactants from different classes often exhibit synergistic effects in reducing surface tension. This means that the total concentration required to achieve a specified surface pressure in the mixture may be lower than that required for an individual surfactant. Such behavior suggests the presence of attractive interactions between the surfactants in the mixed monolayer.

$$\frac{{\left(x\right)}^{2}\ln\left(\frac{aC_{12}}{xC_{1}^{0}}\right)}{{\left(1-x\right)}^{2}\ln\left(\frac{\left(1-a\right)C_{12}}{\left(1-x\right)C_{2}^{0}}\right)}=1$$

$$\beta^{S}=\frac{\ln\left(\frac{aC_{12}}{xC_{1}^{0}}\right)}{{\left(1-x\right)}^{2}}$$

In the formula, α and x represent the molar fraction of surfactant 1 in the total surfactant and the mixed monolayer, respectively. C_01_, C_02_, and C_12_ refer to the concentrations of surfactant 1, surfactant 2, and their binary mixture, respectively, needed to reach the specified surface pressure. Typically, the βs value for non-ideal mixed systems exhibiting synergy is negative, with a greater negative value indicating a more pronounced synergistic effect.

From Table 1, it can be observed that, compared with AES and SMCT, CAB exhibits a stronger interaction with the three main surfactants. The interaction parameter β_s_ for the combination of SLG and CAB reaches −2.822, indicating that SLG and CAB have formed a specific mode of interaction. In contrast, AES shows weak interactions with the three surfactants, while SMCT’s interaction cannot be calculated, likely due to its lack of interaction as indicated by its foam behavior.

**TABLE 1:** The β^s^ value of surfactant interaction in each mixed system.

System	Cmc/mol·L^−1^	γcmc/mN·m^−1^	β^s^	System	cmc mol·L^−1^	γcmc/mN·m^−1^	β^s^
SLS(1)	1.65*10^–3^	23.122	−1.542	LA(1)	1.6*10^–3^	22.521	−2.137
SLS(2)	2.25*10^–3^	23.985	−0.233	LA(2)	2.14*10^–3^	23.621	−0.895
SLS(3)	6.11*10^–3^	22.659	---	LA(3)	7.736*10^–3^	21.0505	---
SLG(1)	9.8*10^–4^	28.923	−2.822				
SLG(2)	2.09*10^–3^	28.899	−0.495				
SLG(3)	5.86*10^–3^	25.902	---				

### 3.3 Molecular dynamic simulation

**FIGURE 7:**
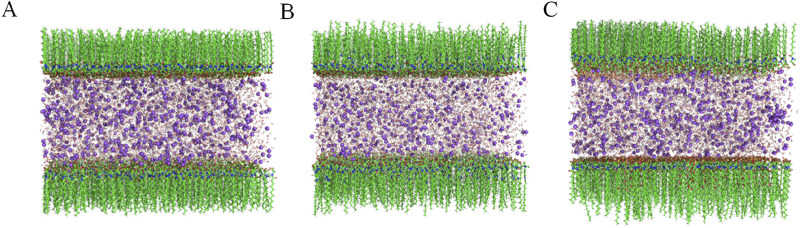
The initial structure of the three surface active sandwich models (green represents carbon atoms, blue represents N atoms, red represents oxygen atoms, yellow represents sulfur atoms, intermediate layer water molecules, and purple represents sodium ions), **(A)** SLG, **(B)** SLG-CAB, **(C)** SLG-AES.

Figure 7 illustrates that the polar atoms (oxygen and sulfur) of the surfactant molecules are oriented towards the central water layer, while the non-polar hydrophobic alkyl groups are oriented away from the water layer, extending into the vacuum region. The molecular lengths follow the order: SLG < CAB < AES. Additionally, SLG and AES are negatively charged ions, whereas CAB is an electrically neutral molecule (Zhang et al., 2022).

#### 3.3.1 Energy analysis

The energy decomposition of SLG molecules in the two systems reveals that the energy of the AES/SLG system is generally higher than that of the CAB/SLG system. In the CAB/SLG system, the average energy is −53.04 kcal/mol, with a minimum value of −70.34 kcal/mol and a maximum value of −31.09 kcal/mol. In contrast, the AES/SLG system has an average energy of −44.97 kcal/mol, with a minimum of −66.19 kcal/mol and a maximum of −21.68 kcal/mol. From this energy analysis, it can be concluded that the CAB/SLG system is more stable than the AES/SLG system (Supplementary Figure S1).

#### 3.3.2 Total hydrogen bond analysis of the system

**FIGURE 8:**
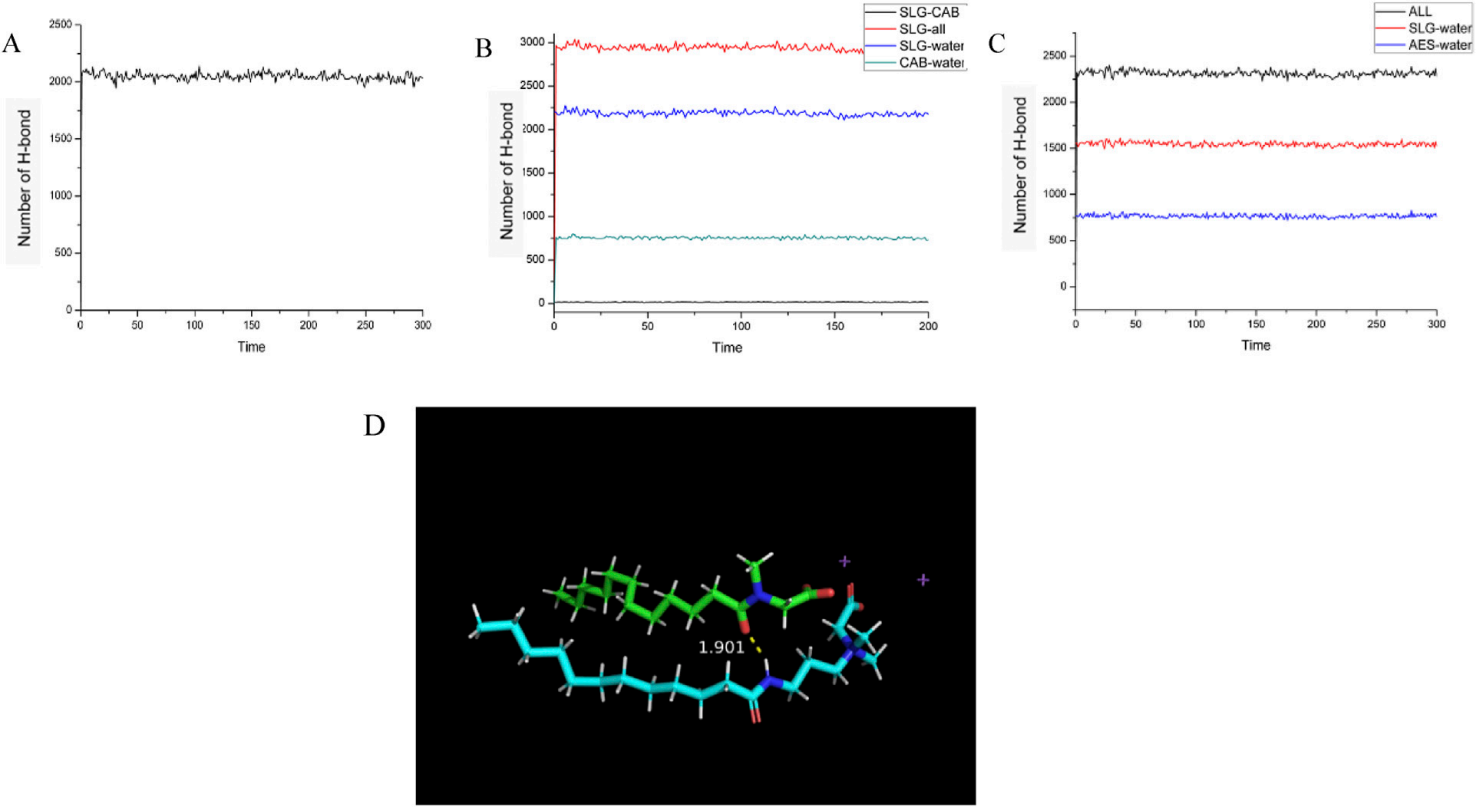
Illustrates the changes in the number of hydrogen bonds during the kinetic simulation process for three systems: **(A)** single-component (SLG) system, **(B)** two-component (CAB/SLG) system, **(C)** two-component (AES/SLG) system. **(D)** Schematic diagram of hydrogen bonding between the two surface active molecules (green for SLG, blue for CAB, and purple for Na ions).

Single-Component (SLG) System (Figure 8A): The SLG system forms hydrogen bonds primarily with water molecules. Once the system reaches equilibrium, the total number of hydrogen bonds fluctuates within a range of approximately 2000.

Two-Component (CAB/SLG) System (Figure 8B): In this mixed system, in addition to the hydrogen bonds between the surfactant molecules (CAB and SLG) and water, hydrogen bonds are also formed between the CAB and SLG molecules themselves. During the 10 ns kinetic simulation, the number of hydrogen bonds between CAB and SLG fluctuates around 15, indicating moderate interaction between the two surfactants.

Two-Component (AES/SLG) System (Figure 8C): The total number of hydrogen bonds in the AES/SLG system is higher than that in the single-component system. This is due to the presence of the sulfonyl group in AES, which contains an additional polar oxygen atom compared to the carboxyl group in SLG. This extra oxygen enhances the ability of AES to form more hydrogen bonds, thereby increasing the overall number of hydrogen bonds in the system.

According to statistical results, SLG is the Acceptor of hydrogen bond and CAB is the Provider of hydrogen bond in all hydrogen bond interactions.

Figure 8D shows the hydrogen bonding between SLG and CAB, where the amino hydrogen of CAB forms a hydrogen bond with the oxygen atom on SLG‘s amide group at a distance of 1.901 Angstrom. And judging from the distance, the effect between the two is strong (<2.0 Angstrom). Hydrogen bonds exist between the parallel superactive molecules, making the sandwich structure formed by superactive molecules more stable.

It is important to note that not all CAB forms hydrogen bonds with SLG. Reason 1: Not all CAB are adjacent to SLG, and hydrogen bonds can only be formed when adjacent distances are close. The ratio of CAB to SLG is 1:2, and not every CAB can bond with SLG. Reason 2: The only potential sites for hydrogen bond formation are amide-oxygen and amino-hydrogen. The basic requirements for forming hydrogen bonds are polar hydrogen and atoms with lone pairs of electrons (O S N). Hydrogen bonds cannot be formed between AES/LA because the above two conditions are not present.

Molecular dynamics simulations confirm the correlation between CAB/SLG/SLS hydrogen-bond networks and enhanced foam lamellar stability, with concurrent observation of energetically favorable membrane configurations.

### 3.4 Foam sebum resistance test

In practical applications, the paramount functionality of foam cleansers resides in their cleansing efficacy, particularly lipid-removal capacity. This study systematically evaluated:

(1) Foam generation capacity of pristine surfactant solutions containing lipid fractions (emulating shampoo application scenarios)
(2) Lipid-elimination performance of generated foams under oily conditions (simulating facial cleansing foam usage)

#### 3.4.1 Degreasing capacity of unformed foam

**FIGURE 9:**
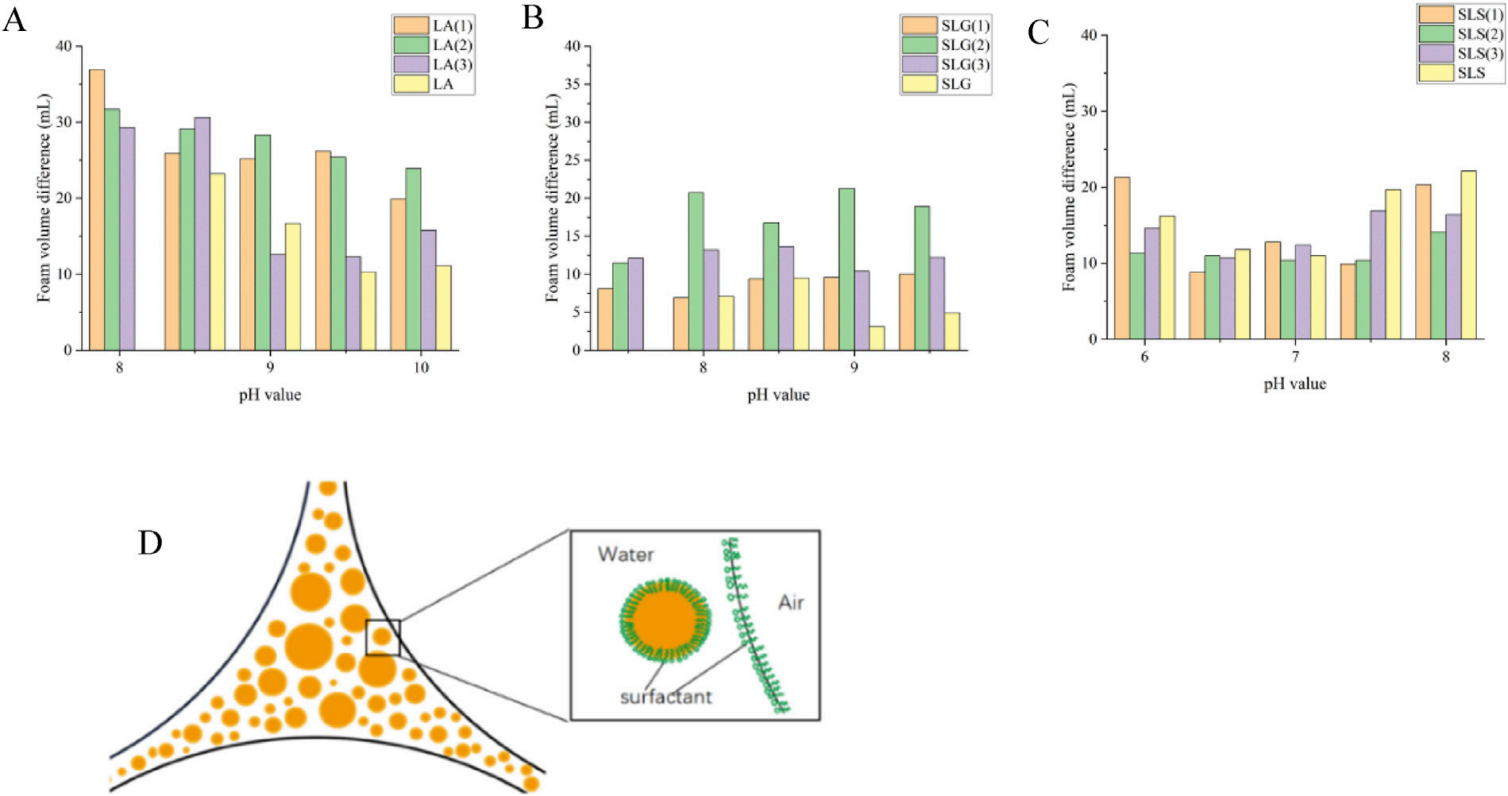
Experimental conditions were: 25°C, 40 mL of foaming solution, with addition of 0.5 wt% synthetic sebum, foaming duration of 48 s. The total foam volume was recorded after completion of the foaming process. **(A–C)** Pure products and different series of Foam valum difference (△V=V _Initial volume_-V _after adding grease_) at different pH: **(A)** LA (1): LA-CAB system, LA (2): LA-AES system, LA (3): LA-SMCT system. **(B)** SLG (1): SLG-CAB system, SLG (2): SLG-AES system, SLG (3): SLG-SMCT system. **(C)** SLS (1): SLS-CAB system, SLS (2): SLS-AES system、SLS (3): SLS-SMCT system; Introduce 0.5 wt% synthetic sebum analog into the baseline surfactant solution, then conduct foaming tests. Compare post-foaming volume with the original system (sebum-free condition). LA Series: At pH 10, pure LA solution exhibits the minimum volume differential (11 mL); at pH 8, LA-CAB formulation shows peak volume variation (36.9 mL). SLG Series:Pure SLG solution demonstrates optimal lipid resistance at pH 9.5 (ΔV = 4.9 mL); SLG-AES blend displays maximum instability at pH 9 (ΔV = 21.9 mL). SLS Series: SLS-CAB system achieves minimum variation at pH 6.5 (ΔV = 8.8 mL); Pure SLS solution shows worst performance at pH 8 (ΔV = 22.1 mL) **(D)** Illustration of the pseudoulsion film between an oil droplet and an aqueous bubble stabilized by the same surfactant.

Figures 3A–D, 9A–C illustrate Δ volume differentials between lipid-containing and lipid-free systems. Greater volume differential indicates inferior lipid resistance of the foam solution, demonstrating heightened susceptibility to lipid interference and failure to maintain original foam volume. Among the three systems, the LA series exhibits the largest foam volume variation (mean differential: 22.86 mL). Superior lipid resistance is achieved exclusively in pure LA solutions under elevated alkaline conditions, where the volume differential remains below 15 mL. SLG demonstrated superior performance with lipid-induced volume decline maintained below 13 mL (notably reaching 3.2 mL at pH 9). Cosurfactants demonstrate no synergistic enhancement in lipid resistance for both LA and SLG systems, instead exhibit pronounced antagonistic effects. But Auxiliary surfactants exhibited selective enhancement solely in SLS systems (CAB showing maximal synergism), partially mitigating lipid-triggered foam collapse—albeit exclusively under mildly alkaline conditions. Figure 9D details the spatiotemporal distribution between oily phases and foam lamellae during post-foaming stages.

#### 3.4.2 Degreasing ability of formed foam

Previous studies (Zhou et al., 2022) have well established the mechanism of oil removal by aqueous foams, which generally involves three key steps: (1) oil droplets entering the gas-liquid interface, (2) oil diffusion across the foam film, and (3) the formation of unstable bridges across the lamella (Qu et al., 2019).

As seen in Table 2, LA foam demonstrates the highest oil removal rate, while the SLG and SLS series show oil removal rates below 80%, with pure SLS foam being almost ineffective in removing oil. This phenomenon is likely due to the size and stability of the foam.

**TABLE 2:** Cleaning efficiency of each series of surfactants.

System	pH	Cleaning effciency/%	System	pH	Cleaning effciency/%
LA	9	80.92%	SLS	7	2.15%
LA(1)		84.69%	SLS(1)		68.27%
LA(2)		80.72%	SLS(2)		52.11%
LA(3)		83.75%	SLS(3)		12.08%
SLG	8.5	33.12%			
SLG(1)		74.74%			
SLG(2)		66.39%			
SLG(3)		47.71%			

Illustration: A specific amount of oil was applied to a glass plate, and foam was sprayed onto the plate. The change in the oil area was recorded using a camera. After the cleaning process, the foam was removed, and the glass plate was dried and weighed. The cleaning efficiency was calculated using the following formula $:Cleaning\;effciency\%=\left(m_{1}-m_{2}\right)/\left(m_{1}-m_{0}\right)*100\%$ (Where m_0_ is the initial weight of the soiled glass plate; m_1_and m_2_ are the weights of the contaminated glass plate before and after cleaning, respectively).

Smaller Bubbles and Oil Removal: Smaller bubbles create more bubble gaps within the same volume, which generates capillary forces that help absorb oil. This imbibition process draws oil into the foam’s boundary layer due to the positive effective surface tension of the surfactant. Furthermore, the lower surface tension of the system facilitates the imbibition of crude oil (Supplementary Figure S2).

LA Foam: The bubble size of the LA system is smaller than that of the pure SLS system. Additionally, the better foam stability of LA reduces the likelihood of foam coalescence and collapse. As a result, the LA foam exhibits a larger capillary effect over time, leading to higher oil removal efficiency.

SLG Foam: The SLG system also produces smaller bubbles compared to the pure SLS foam. However, due to its poorer foam stability, SLG foam tends to gather and rearrange. Despite this, the frictional effect generated during this rearrangement helps remove oil from the solid surface. The central area of the foam demonstrates oil removal, which highlights the friction-induced movement of the unstable foam (Supplementary Figure S3).

## 4 Conclusion

This systematic investigation establishes the structure-property relationships governing foam dynamics and lipid removal efficacy in amino-acid-derived surfactant blends, elucidating the regulatory roles of carboxamide groups, branched-chain moieties, and synergistic effects on practical performance and consumer perception. Key findings from dynamic foam analysis, molecular dynamics (MD) simulations, and lipid resistance assays are summarized as follows:

While the sodium Lauric acid (LA) system exhibited superior apparent delipidation efficiency (80.92%) attributable to ultrafine bubble architecture (<90 μm), its alkaline formulation (pH 9.3 ± 0.2) risks compromising epidermal barrier integrity. Sodium N-lauroyl sarcosinate (SLS) and sodium N-lauroyl glycinate (SLG) demonstrated pH-responsive foam dynamics governed by carboxylic acid protonation equilibria. Branching and carboxamide group introduction improved cutaneous affinity but compromised foam kinetics (nucleation time >35 s, half-life reduction). This paradox was resolved through co-formulation with cocamide propyl betaine (CAB) and sodium lauryl ether sulfate (AES). SLG-CAB blends in pH 7-8 generated dense foams (mean diameter≈95 μm, T_fls_ 50% > 5 min) delivering dual sensory attributes of velvety tactile perception and prolonged creaminess. CAB enhanced synergistic performance (β_s_ = −2.822) via charge complementarity, accelerating foam generation kinetics (35 s→20 s/100 mL at pH 8.5). MD simulations revealed hydrogen-bond networks (SLG-CAB d = 1.901 Å) and interfacial energy (ΔE = −44.97 kcal/mol) that stabilized lamellar structures through increased film viscosity, effectively suppressing Ostwald ripening. Under lipid-loaded conditions (0.5% artificial sebum), SLG-CAB maintained 74.74% lipid solubilization efficacy versus 33.12% for pristine SLG. SLS-CAB formulations optimized foam performance at physiologically favorable pH 6.5–7.5 (foam volume 120 mL, Tfls 50% > 5 min, lipid removal 68.27%), addressing the mildness-efficacy tradeoff.

This study establishes a quantitative correlation model encompassing phase behavior-foam dynamics-consumer perception, providing theoretical foundations for formulation optimization strategies of amino-acid-based surfactants via supramolecular state modulation. The methodology proves particularly applicable to developing facial cleansers and body wash products that simultaneously address pH compatibility (5.5–8.5), sensory excellence (>110 mL foam volume, <95 μm bubble diameter), and epidermal barrier protection.

## Data Availability

The original contributions presented in the study are included in the article/Supplementary Material, further inquiries can be directed to the corresponding author.
