# Supplementary figures and images for: Impact of molecular structures of lauroyl glycine derivatives on foaming properties
Source: Front Chem. 2025 Jun 24;13:1563560. doi: 10.3389/fchem.2025.1563560 (PMC12235571; doi:10.3389/fchem.2025.1563560)

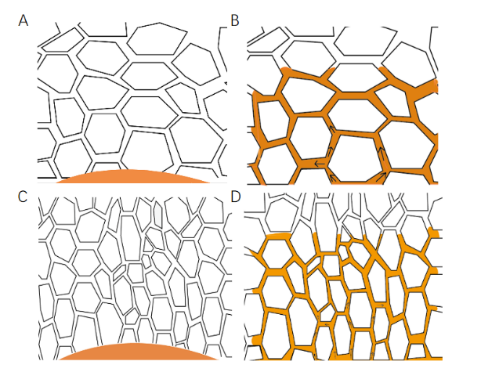

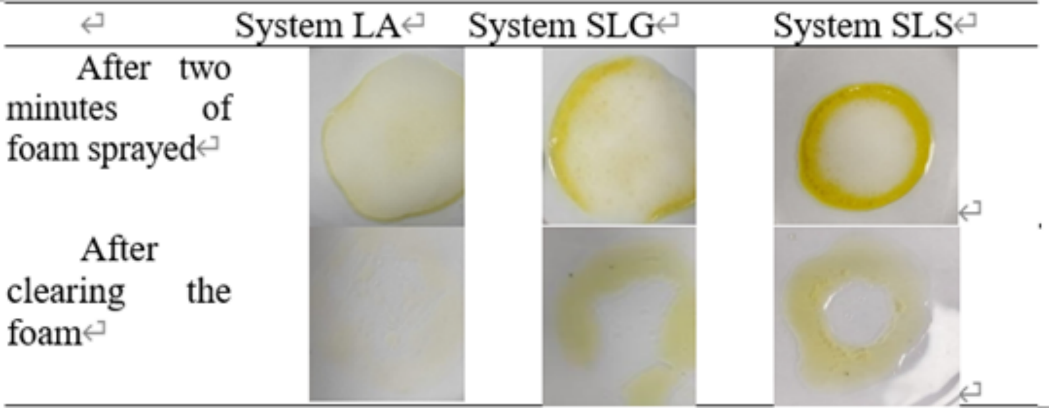

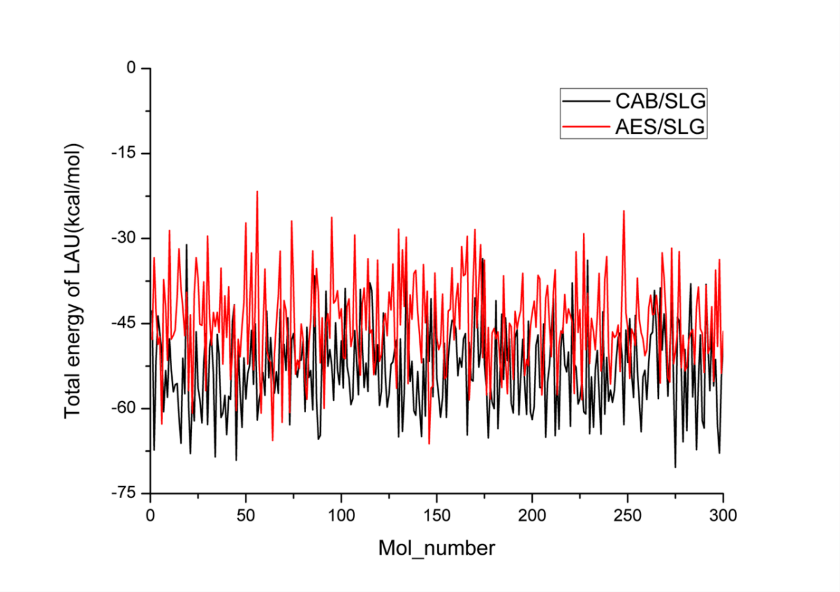


Supplementary Picture.3

Supplementary Picture.1

Supplementary Picture.2

Supplement: Supplementary file 1 [file DataSheet1.docx]
